# What Do We Know About Education in Colorectal Cancer Prevention?—Survey Among 1130 Medical Students

**DOI:** 10.1007/s13187-015-0967-0

**Published:** 2015-12-26

**Authors:** Łukasz Pietrzyk, Anna Torres, Marta Denisow-Pietrzyk, Kamil Torres

**Affiliations:** 1 Department of Didactics and Medical Simulation, Medical University of Lublin, Jaczewskiego 4, 20-094 Lublin, Poland; 2 Department of General and Minimally Invasive Surgery, 1st Military Clinical Hospital in Lublin, Lublin, Poland; 3 Department of Human Anatomy, Medical University of Lublin, Lublin, Poland

**Keywords:** Colorectal cancer, Oncology, Medical education, Medical student, Questionnaire

## Abstract

Colorectal cancer (CRC) is a global public health problem. The degree of knowledge on CRC among medical students, future physicians, brings essential implications for their patients. Therefore, to acquire information about students’ knowledge on CRC, a questionnaire survey was conducted in Medical University of Lublin, Poland, with a representative sample of 1130 medical students (basic vs. clinical 552 vs. 578; male vs. female 442 vs. 688). The questionnaire was anonymous and designed in a four-section scheme (CRC risk factors, CRC prevention, CRC symptoms, CRC screening methods). There was a significant difference in the percentage of correct answers between basic and clinical level groups (*P* = 0.034). In general, clinical students had higher scores for their knowledge regarding CRC. Gender impacted the students’ CRC knowledge to a lesser degree; however, if the difference was revealed, female students were more aware about CRC issues (*P* = 0.045). We found several important deficits in students’ knowledge on CRC. These findings should motivate the oncology education coordinator as well as our teachers to introduce innovations in education methods and training environments to enable students to gain necessary knowledge and acquire the skills and competencies that would help them to function as physicians.

## Introduction

Colorectal cancer (CRC), with nearly 1.4 million newly diagnosed cases in 2012, is the third most common neoplasm worldwide [1]. As in other countries, in Poland, an increasing trend in new CRC incidents is noted. For example, in 2010, 15,800 new cases have been diagnosed (8700 in males and 7100 in females); thus, an approximately fourfold increase has been evidenced since 1980 [2]. Heredity, gender, or ethnic as well as environmental factors (family status, lifestyle, smoking, low-fiber diet, overweight, and obesity-related syndromes) appear to be among the important factors of CRC risk, as reviewed by Pietrzyk et al. [3]. In Poland, the 5-year survival rate (below 25 %) is one of the lowest in the world [2]. The possible cause is due to a poor general health care system. Moreover, the National Colorectal Cancer Screening Program launched in October 2000 is deeply unpopular, presumably due to both the low health awareness of the Polish society and the inadequate education of physicians [4]. To decisively counteract this, the National Cancer Strategy (NCS) has been developed for 2015–2024 as an initiative of the Polish Society of Oncology, other scientific associations, and the Ministry of Health. The goals include a wide range of issues, i.e., coordination in defining and maintaining up to date all the diagnostic and therapeutic standards for all cancer types as well as training of health care professionals and promotion of scientific research. Building consciousness of well-trained multidisciplinary medical teams is an important tool in NCS, and the realization of prevention and CRC screening programs is a part of it. The CRC prevention strategy involves also improvement of the current medical education, starting from the academic degree [5]. It may sound unconvincing, but the development of the medical education in Eastern European countries, including Poland, has been affected by some historical determinants (the impact of the Cold War) and related to the heritage of communism, e.g., the lack of funds that hindered modern education methods. Since the 1990s, the transformation date, medical education is being constantly developed in Poland [6, 7]. Medical education programs, including oncology education, were reviewed and accepted by the Ministry of Health. Integrated studies of medicine encompass basic courses and clinical courses. The oncology curriculum was based on the experience and recommendations of the European Association for Cancer Education (EACE) and the Union for International Cancer Control (UICC) [4]. At all medical universities, oncology is incorporated into the teaching courses in basic and clinical medicine programs with core lectures and seminars and the minimum number of classes devoted to oncology is 70 [7, 8]. Academic teachers are supervised by an oncology education coordinator, who was appointed since 2010 [9]. The main hopes for improvement are the increase (i) in the number of integrated oncology units and (ii) in the number of academic teachers representing different branches of oncology [8]. However, despite the importance of colorectal cancer, the knowledge on CRC prevention issues is reported to be insufficient among medical students [8, 10]. Therefore, we designed a questionnaire survey for medical students to identify gaps in their knowledge about (i) CRC risk factors, (ii) CRC prevention, (iii) CRC-associated symptoms, and (iv) CRC screening program in Poland. Information about knowledge deficit among medical students could help teachers to develop better teaching strategies and to prepare a more creative curriculum in order to improve students’ learning outcomes.

## Methods

### Study Population and Procedures

A cross-sectional study was conducted between May and June 2015 among 1130 first- to sixth-year medical students of the Medical University of Lublin, Poland. The study population consisted of a basic-level group, which included first-, second-, and third-year medical students, whereas fourth-, fifth-, and sixth-year medical students were enrolled to a clinical-level group (basic vs. clinical group 552 vs. 578 students). Among the total participants, 442 were males and 688 females. Participants were randomly selected. Prior to the questionnaire distribution, all students selected to the survey were informed by the research team about the objectives of the study and on a voluntary basis accepted to participate in the survey. Participants completed a hard copy version of the questionnaire. The survey questions were developed basing on literature review and revised by a research team, including clinicians. The questionnaire was designed in four sections to provide information about medical students’ knowledge of (I) CRC risk factors, (II) CRC prevention, (III) CRC-related symptoms, and (IV) CRC screening methods. The participants were asked to answer the questionnaire completely anonymously. Before the study has started, the questionnaires were distributed to a group of 15 students who were further excluded from the study population. The aim of this pilot study was to improve the questionnaire. The questionnaires were handed and collected in the seminar rooms by the technician staff to avoid the involvement and eventual influence of researchers on the process of data collection.

### Statistical Analysis

Data was analyzed using descriptive statistics and presented as percentages. The chi-squared test was used to compare proportions of answers between groups of respondents: (i) basic- vs. clinical-level students and (ii) male vs. female students. Differences were considered as statistically significant at *P* < 0.05. Statistical analysis was performed using SPSS Version 16.0 (SPSS Inc., Chicago, IL, USA).

## Results

One thousand and one hundred-thirty students answered the questionnaire. The respondents’ mean age was 20.8 years (range 19–25 years) and 60.9 % were females. The factor that influenced the students’ knowledge was the course level, and there was a difference between different basic and clinical groups for most of the analyzed knowledge criteria. In general, clinical students had higher scores for their knowledge on colorectal cancer risk factors, prevention, and screening compared with basic-level students (*P* = 0.034). Gender impacted the students’ CRC knowledge to a lesser degree. However, if there were significant differences between males and females, consciousness about CRC was more pronounced among women (*P* = 0.045).

Most (67–96.7 %) of the participants were aware of age as a factor increasing the risk of CRC; however, consciousness was significantly higher among the clinical-level group of students (Table 1).

**Table 1:** The percentages of medical students who agreed with statements regarding age as a risk factor for colorectal cancer

Age group as risk factor	Basic level	Clinical level	*P*	Male	Female	*P*	Total population
20–30	1.0	0.1	0.042	0.5	0.6	0.031	1.1
30–40	9.6	7.1		6.5	10.2		16.7
>50	32.5	49.7		30.4	51.8		82.2

*P* value according to chi-squared test for the difference between basic and clinical groups and between male and female groups

A significant proportion of medical students (70–80 %) were informed that a fat-rich and low-fiber diet, substances produced during frying, ulcerative colitis, and a family history of the disease are among the important CRC risk factors (Table 2). In general, 88.4 % of basic-level and 94.3 % of clinical-level students, 91.4 % on average have identified clearly that colon polyps are highly predictive of CRC. The participants tend to lack awareness that diabetes mellitus (only 15.3 % of the total students), coffee drinking (23.1 % of the total students), and a carbohydrate-rich diet (36.8 % of the total students) may increase the chance of developing CRC. Only 53.4 % of the total study group declared that Crohn’s disease is associated with colon cancer. Obesity was identified among the risk factors by 66.8 % of the participants.

**Table 2:** The percentages of medical students who agreed with statements regarding the risk factors for colorectal cancer

Risk factors for colorectal cancer	Basic level	Clinical level	*P*	Male	Female	*P*	Mean for total population
Fat-rich diet	80.3	87.1	0.044	83.3	84.0	ns	83.7
Low-fiber diet	76.8	93.4	0.032	82.5	87.0	0.042	85.3
Carbohydrate-rich diet	29.7	43.8	0.021	38.5	34.4	ns	36.8
Substances produced during frying	77.2	82.4	0.036	80.4	79.5	ns	79.9
Coffee drinking	29.2	17.4	0.012	24.5	22.4	ns	23.1
Smoking	68.3	79.0	0.029	73.6	74.1	ns	73.8
Obesity	71.9	61.9	0.023	64.4	68.4	ns	66.8
Crohn’s disease	48.4	58.3	0.018	52.1	54.4	ns	53.4
Ulcerative colitis	84.6	78.4	0.026	81.1	81.9	ns	81.6
Colon polyps	88.4	94.3	0.037	92.2	90.9	ns	91.4
Diabetes mellitus	19.2	11.6	0.035	17.0	14.3	ns	15.3
Family history of cancer	74.6	85.2	0.041	77.1	82.0	0.019	80.2

*P* value according to chi-squared test for the difference between basic and clinical groups and between male and female groups

*ns* difference not significant

A significant proportion of medical students was informed that physical activity (83.6 % of total), avoidance of a high-fat diet (83.5 %), as well as a diet composed of fruits and vegetables (83.5 %) decrease in average the risk of CRC (Table 3). Interestingly, only 42.3 % of the respondents recognized reduction of calorie intake among the specific prevention methods. The awareness that smoking cessation and control of alcohol consumption may prevent CRC disease was low, only 73.1 and 68.6 % of the total participants, respectively.

**Table 3:** The percentages of medical students who agreed with statements regarding the prevention of colorectal cancer

Preventive factors	Basic level	Clinical level	*P*	Male	Female	*P*	Mean for total population
Increase the intensity and regularity of physical activity	61.6	65.3	ns	59.4	66.1	0.042	63.6
Avoid high-fat diet	78.8	87.9	0.038	84.4	82.9	ns	83.5
Increase the consumption of fruits and vegetables	77.5	89.1	0.021	82.8	83.9	ns	83.3
Reduce calorie intake	36.1	48.3	0.018	36.6	46.0	0.022	42.3
Avoid excess alcohol	66.8	67.6	ns	68.6	65.7	ns	67.2
Stop smoking	69.0	80.2	0.038	73.1	75.9	ns	74.6

*P* value according to chi-squared test for the difference between basic and clinical groups and between male and female groups

*ns* difference not significant

Regarding the students’ knowledge on colorectal cancer signs and symptoms, the majority of students properly indicated rectal bleeding (79.9 %) and fresh blood in stool (80.7 %) (Table 4). However, relatively few responders pointed out change of stool consistency (52.5 %), lack of appetite, weakness, and fatigue (41.8 %) among the meaningful CRC signs. Black stool was identified as a CRC-specific syndrome by 30.7 % of the study groups. Interestingly, awareness of the link between black stool and CRC was significantly higher in the basic-level group of students.

**Table 4:** The percentages of medical students who agreed with statements regarding the symptoms of colorectal cancer

Symptoms of colorectal cancer	Basic level	Clinical level	*P*	Male	Female	*P*	Mean for total population
Rectal bleeding	77.3	82.4	0.037	74.8	83.0	0.039	79.9
Fresh blood in stool	75.5	85.7	0.029	80.0	81.1	ns	80.6
Black stool	33.9	20.0	0.035	24.2	30.1	0.022	27.0
Change in bowel habits, including diarrhea and constipation	54.8	70.5	0.018	57.3	66.4	0.028	63.0
Change of consistency of stool	42.5	61.9	0.002	50.2	53.9	ns	52.5
Persistent abdominal discomfort	68.8	60.7	0.036	65.8	63.8	ns	64.6
Unexplained weight loss	60.1	87.1	0.004	70.8	75.7	ns	73.9
Lack of appetite, weakness, and fatigue	35.6	47.6	0.041	41.5	42.0	ns	41.8

*P* value according to chi-squared test for the difference between basic and clinical groups and between male and female groups

*ns* difference not significant

Regardless of the academic level, medical students are correctly informed that a colorectal cancer screening program has been incorporated in Poland (Tables 5 and 6). However, their knowledge about the criteria for an individual selection to the program was very poor. Generally, 58.7 % of the participants agreed age was among the inclusion criteria, but only 25.1 % of the total students recognized correctly all inclusion criteria for recruitment to the CRC screening program. Majority of students (87.3 %) had knowledge that colonoscopy is offered as a free screening program to prevent colorectal cancer; clinical students were more conscious that colonoscopy is used as the primary screening modality in the national CRC prevention program (81.1 % basic vs. 93.3 % clinical). There were 12.6–39.4 % of the total students who were false-positives about tools employed in the program. Significantly more clinical- than basic-level students (61.9 vs. 20.1 %) were conscious where the screening program is available.

**Table 5:** The percentages of medical students who agreed with statements regarding the inclusion criteria for colorectal cancer screening programs in Poland

Criteria	Basic level	Clinical level	*P*	Male	Female	*P*	Mean
People aged 50 to 66 years with no clinical suspicion of CRC	39.9	76.7	0.003	52.7	62.4	0.026	58.7
People aged 40 to 70 years with no clinical suspicion of CRC	24.6	7.6	0.002	18.2	14.5	ns	15.9
People aged 40–49 with a family cancer history	32.4	46.7	0.023	31.4	44.6	0.037	39.7
People aged 50 to 66 years (for people with a family cancer history, age limit was <40 years) with no clinical symptoms of CRC	13.3	36.2	0.028	18.7	29.0	0.014	25.1
There is no such program in Poland	5.6	2.8	0.043	7.6	2.1	0.041	4.2

*P* value according to chi-squared test for the difference between basic and clinical groups and between male and female groups

*ns* difference not significant

**Table 6:** The percentages of medical students who agreed with statements regarding the methods used in screening programs for colorectal cancer in Poland

Screening method	Basic level	Clinical level	*P*	Male	Female	*P*	Mean for total population
Colonoscopy	81.1	93.3	0.031	82.3	90.3	0.039	87.3
Blood CEA level	20.0	10.2	0.038	12.9	18.4	0.041	15.0
Fecal occult blood test	38.8	39.8	ns	41.8	37.9	ns	39.4
Digital rectal examination	41.9	34.8	0.042	37.6	38.8	ns	38.3
Rectoscopy	13.8	11.4	ns	14.9	11.2	ns	12.6
There is no such program in Poland	4.7	3.3	ns	6.9	2.3	0.044	4.0

*P* value according to the chi-squared test for the difference between basic and clinical groups and between male and female groups

*ns* difference not significant

## Discussion

Emphasis on medical education is an integral part of the strategy for the decrease of CRC incidents [4]. Therefore, we conducted the survey in order to evaluate the awareness of our medical students on CRC risk factors, prevention, symptoms, and related issues of colorectal cancer diagnosis and national screening modalities. The present study acquired information that would guide educators about knowledge deficit among medical students. In Polish universities, a uniform curriculum in academic oncology has been introduced since 2010 [7, 9]. Therefore, despite the fact that our study is limited to a single university, it has a relatively large sample size and we expect the study will serve the academic body to keep and/or improve medical education standards to meet the public interest in respect of prevention and quick diagnosis of CRC.

We found that the knowledge regarding CRC increased in the clinical years, as evidenced by the increase in correct answers between basic and clinical levels. This suggests that the environment to which our students are exposed is relevant for development of knowledge. This also indicates that our clinical classes (observation of clinical practices, contact with patients) are particularly important in developing CRC knowledge. Many studies reported that practical classes are more effective for medical education and students’ achievements than theoretical classes [10–13].

Regardless of the academic levels, our results demonstrated gaps in different aspects of CRC knowledge among students. These results are in agreement with published literature regarding cancer education among medical students. Multiple studies have revealed that cancer education is inadequate, fragmented, and poorly coordinated at medical universities [13–15]. With this in mind, we have to consider which elements of our education programs clearly need standards improvement in terms of CRC risk factors, prevention, as well as symptoms and national screening programs.

The first part of the questionnaire was developed to evaluate knowledge on CRC risk factors, and the second part focused on CRC prevention methods. We expected all students to achieve at least 80 % of the scores in the identification of CRC risk and prevention factors. High scores were achieved in identifying some risk factors for CRC; these included a fat-rich diet, a low-fiber diet, tobacco use, and a family history of CRC. However, low scores were observed when identifying risk factors such as a carbohydrate-rich diet, coffee drinking, or diabetes mellitus. The students’ knowledge about the potential obesity contribution to the prognosis of colorectal cancer is also inadequate; almost 36 % of the students did not link obesity with an increased incidence of CRC. The WHO formally recognized obesity as a global epidemic; therefore, CRC prevention strategies will require providing patients with sufficient information on the relationship between obesity and the risk of CRC development [3, 16]. Therefore, our students’ awareness of the obesity-CRC relationship needs to be rapidly changed. It has been suggested that cancer prevention education in medical schools is essential for medical students, tomorrow’s physicians [14]. Physicians play a crucial role in promoting healthier lifestyles, advise how to prevent disease, and can considerably impact on the patients’ attitudes towards CRC [17]. Medical students’ sufficient knowledge concerning protective topics might lead to a prospective increase of social consciousness that CRC development might be stopped by patients’ own actions regarding lifestyle. In this respect, students’ knowledge about potentially modifiable factors (e.g., obesity, drinking alcohol, smoking) is fundamental and should be sufficiently monitored. At Polish medical universities, efforts are being made to give information about the social factors in cancer etiology since the first year of medical education [9]; therefore, attempts are needed to improve training methods in oncology-related classes.

In our study, a relatively low percentage of students was conscious that a reduction in daily caloric intake and avoidance of alcohol might significantly reduce CRC risk. High-calorie foods as well as alcohol consumption are a part of “westernization” of lifestyle [16]. Harmful or hazardous consumption of alcohol beverages has been identified as being a problem among medical students [18–20]. It is possible that the students who participated in our survey treat fatty food and alcohol consumption as integral parts of life and do not recognize them as factors that cause health problems. A poor level of knowledge towards the relationship between lifestyle and colorectal cancer was reported among medical students in Mexico [12] or in Malaysia [21]. Deficits in knowledge were also reported among medical students in the USA [17]. Therefore, preventive medicine was implemented in several universities and it is believed that education regarding healthy lifestyle habits present in medical curricula would be an advantage and would help physicians to keep their patients healthy [10, 17].

We found it very disturbing that almost 6 % of our clinical-level students and almost 12 % of basic-level students were not aware that colon polyps are among the potential risks of colon cancer. The students’ awareness of easy to detect and remove non-cancerous polyp abnormalities before they have the chance to turn into cancer [3, 4] should not be ignored. Medical students as well as physicians may have a great impact on patients’ motivation to remove polyps, reducing the risk of dying from colorectal cancer [11–13]. Knowledge on preventive behaviors may also improve attitude, disbelief, and misunderstanding and consequently improve healthy living and enhance screening practices.

The third part of our questionnaire was designed to assess the students’ knowledge in relation to CRC symptoms. The present study revealed insufficient knowledge towards colorectal signs. For example, very low scores were observed in relation to black stool and changes of stool consistency. The gaps in knowledge about CRC-specific symptoms were reported for Greek and American students [15, 22]. Teaching the disease symptoms is an integral part of medical education, and emphasis on cancer symptoms in the medical curriculum possibly would result in early cancer detection and in consequence could hinder the cancer disease [22, 23]. Several authors have pointed out that fragmented knowledge on cancer symptoms is related to a low level of medical students’ exposure to patients with cancer [9, 10, 14]. In Poland, attempts are made to increase the knowledge of medical students regarding oncology by launching, by the Polish government, the “National Program for Combating Neoplastic Diseases” incorporated since 2005. One of the advantages of the program is the increase of clinical classes in oncology units at patients’ beds [9]. However, our survey indicated that we need to develop an algorithm to achieve students’ knowledge efficiency.

The fourth part of the questionnaire was designated to assess students’ attitude towards CRC screening methods. Fatalistic attitudes have been identified among students for screening methods in the USA [14]. The students’ and physicians’ approach to CRC screening modalities, especially colonoscopy, may have a great impact on the patients’ opinion and motivation to undergo colonoscopy [15]. It is encouraging that a remarkably high percentage (81–93 %) of our students reported colonoscopy among the screening methods used in national CRC prevention programs. However, in terms of access to the program and screening tests for CRC, a high proportion of participants were not able to indicate how to be involved in the program. Also, our students were not aware of the selection criteria for CRC screening programs.

The correct response rate among female students was higher than the response among males. In literature, numerous gender differences are discussed regarding work organization and types of care rendered by male and female medical students and doctors [24]. The female gender is usually associated with a stronger intention towards knowledge improvement as well as in continuing medical education [25].

Undoubtedly, all medical students have to gain essential knowledge about CRC. However, the most important questions are what should be learned at the basic and clinical levels and what are the most efficient teaching methods [9, 26]. Most likely, we need to develop the instrument for improvement in curricular modules in subsequent years to raise the awareness of CRC among medical students. There are many debates in the medical education literature regarding how to pass the knowledge best and assess students’ skills and competencies, and it is accepted that improving the quality of assessments in medical programs is beneficial to students and their patients [11–17, 26, 27]. It is certainly the coordinator of academic medical programs who should be responsible for filling gaps in the program. The primary objective of medical education is to provide basic knowledge necessary to build on during postgraduate training. However, the essential problem in an academic medical education in Poland is that existing oncology units are seldom integrated [9]. We assume that the undergraduate curriculum should include more oncology-related courses with emphasis on preventive medicine and hands-on courses that would allow students to familiarize symptoms and screening tools.

Despite the weakness and gaps in our students’ knowledge, we look forward to the future with a lot of optimism. We hope the survey will enable us to give a new momentum to raise educational scores. Several universities have modified their undergraduate programs in oncology and teaching methods, introducing up-to-date methods, including computer learning and simulation techniques [13, 14, 17, 26, 27]. Our university is in a group of Polish medical universities which improve undergraduate oncology education [9].

To summarize, improving the quality of oncological education is a long-term process. Our data would suggest that there remain areas that need to be addressed and that there are several implications for future curriculum development in regard to colorectal cancer education particularly on risk factors, prevention, symptoms, and screening methods.
